# *N*,*N*-Bis(2-pyridylmeth­yl)-*tert*-butyl­amine

**DOI:** 10.1107/S1600536809001366

**Published:** 2009-01-28

**Authors:** Allen Mambanda, Deogratius Jaganyi, Kirsty Stewart

**Affiliations:** a School of Chemical and Physical Sciences, University of KwaZulu–Natal, Scottsville 3209, South Africa

## Abstract

In the title compound, C_16_H_21_N_3_, the dihedral angle between the two pyridine rings is 88.11 (9)°. In the crystal, mol­ecules are linked through inter­molecular C—H⋯π inter­actions, forming a layer expanding parallel to the (10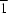) plane.

## Related literature

For related compounds, see: Mambanda *et al.* (2007 ▶); Foxon *et al.* (2007 ▶); Fujihara *et al.* (2004 ▶); Munro & Camp (2003 ▶). For metal complexes with the title compound as a ligand, see: Fujii *et al.* (2003 ▶); Lee & Lippard (2002 ▶); Mok *et al.* (1997 ▶). For the metal complex with *N*,*N*-bis­(2-pyridylmeth­yl)ethyl­amine as a ligand, see: Pal *et al.* (1992 ▶).
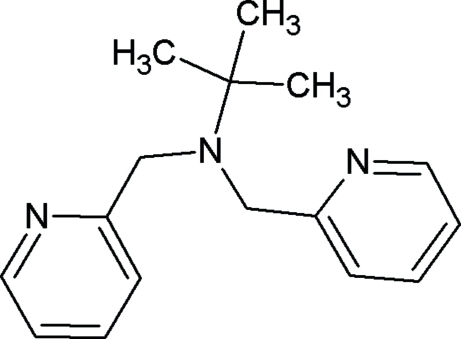


## Experimental

### 

#### Crystal data

C_16_H_21_N_3_
*M* *_r_* = 255.36
Monoclinic, 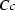

*a* = 6.1808 (3) Å
*b* = 17.9502 (8) Å
*c* = 13.7079 (6) Å
β = 100.239 (4)°
*V* = 1496.62 (12) Å^3^
*Z* = 4
Mo *K*α radiation
μ = 0.07 mm^−1^
*T* = 293 (2) K
0.50 × 0.50 × 0.30 mm

#### Data collection

Oxford Diffraction Xcalibur2 CCD diffractometer
Absorption correction: multi-scan (**CrysAlis RED**; Oxford Diffraction, 2008 ▶) *T*_min_ = 0.967, *T*_max_ = 0.980
7475 measured reflections
2392 independent reflections
2024 reflections with *I* > 2σ(*I*)
*R*_int_ = 0.012

#### Refinement

*R*[*F* ^2^ > 2σ(*F* ^2^)] = 0.043
*wR*(*F* ^2^) = 0.119
*S* = 1.02
2392 reflections
175 parameters
2 restraints
H-atom parameters constrained
Δρ_max_ = 0.21 e Å^−3^
Δρ_min_ = −0.22 e Å^−3^

### 

Data collection: *CrysAlis CCD* (Oxford Diffraction, 2008 ▶); cell refinement: *CrysAlis RED* (Oxford Diffraction, 2008 ▶); data reduction: *CrysAlis RED*; program(s) used to solve structure: *SHELXS97* (Sheldrick, 2008 ▶); program(s) used to refine structure: *SHELXL97* (Sheldrick, 2008 ▶); molecular graphics: *ORTEP-3* (Farrugia, 1997 ▶); software used to prepare material for publication: *WinGX* (Farrugia, 1999 ▶).

## Supplementary Material

Crystal structure: contains datablocks I, global. DOI: 10.1107/S1600536809001366/is2355sup1.cif

Structure factors: contains datablocks I. DOI: 10.1107/S1600536809001366/is2355Isup2.hkl

Additional supplementary materials:  crystallographic information; 3D view; checkCIF report

## Figures and Tables

**Table 1:** Hydrogen-bond geometry (Å, °) *Cg*1 and *Cg*2 are the centroids of the N2/C3–C7and N3/C9–C13 rings, respectively.

*D*—H⋯*A*	*D*—H	H⋯*A*	*D*⋯*A*	*D*—H⋯*A*
C7—H1⋯*Cg*2^i^	0.93	2.97	3.819 (2)	153
C15—H16⋯*Cg*1^ii^	0.96	2.94	3.836 (2)	156

Symmetry codes: (i) 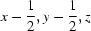; (ii) 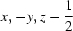.

**Fig. 1.**
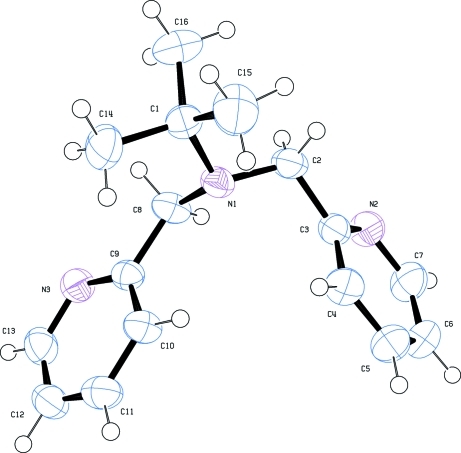
The molecular structure of the title compound, showing 50% probability displacement ellipsoids and atomic numbering.

**Fig. 2.**
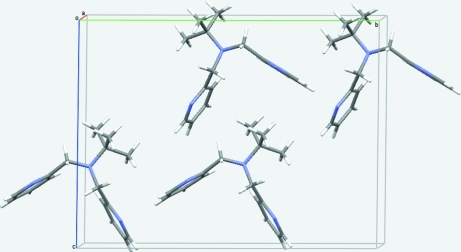
Unit cell packing diagram of the title compound, viewed along the a axis.

## supplementary crystallographic information

### Comment

The title compound is a versatile tridentate N-donor ligand, and it (Fujii *et al.*, 2003; Lee & Lippard, 2002; Mok *et al.*, 1997) and its analogue, *N, N*-bis(2-pyridylmethyl)ethylamine (Pal *et al.*, 1992) have been used extensively in metal coordination. The related crystal structures of symmetrical bis(tridentate) ligands have been reportd (Mambanda *et al.*, 2007; Foxon *et al.*, 2007; Fujihara *et al.*, 2004).

The crystal structure of the title compound (Fig. 1) shows that the three nitrogen atoms (one *sp*^3^ and two pyridine *sp*^2^) are not suitability orientated for pincer-like coordination to a metal. Rotation about the C2—C3 and C8—C9 bonds are required for that to occur. The relative orientation of the two pyridine rings is reflected in a dihedral angle between their mean planes of 88.11 (9)°, clearly this angle would have to change were the ligand to bind to a metal centre. The steric influence of the bulky *tert*-butyl group is reflected in the C1—N1—C2 and C1—N1—C8 angles [113.90 (13) and 115.34 (13)°, respectively], being larger than the C2—N1—C8 angle of 110.08 (13)°. The methylene groups of the structure all adopt the expected staggered (lowest energy) conformation (Munro & Camp, 2003). The pyridyl ring containing atom N2 is orientated at 18 (1)°  relative to the mean plane of the *N*-*tert*-butyl group (plane through C16—C1—N1), whilst that containing N3 is orientated at 74 (1)°.

There are no short van der Waals contacts less than the sum of the van der Waals radii in this system, reflected in the loose packing, however weak (possibly stabilizing) C—H···π intermolecular interactions do occur. The metrics of such interactions reflect a T-shaped, edge-to-face geometry. Specifically, let us define *Cg*1 as the centre of gravity of the pyridyl N2/C3–C7 ring and *Cg*2 the centre of gravity of the pyridyl N3/C9–C13 ring. A C—H···π interaction with a separation of 2.97 (1) Å exists between C7—H1 from the pyridyl ring containing atom N2 and *Cg*2 of neighbouring symmetry related molecule [symmetry code: (i) *x* - 1/2, *y* - 1/2, *z*] (Table 1). A similar C—H···π interaction with a separation of 2.94 (1) Å exists between C15—H16 from one of the methyl groups of the *tert*-butyl moiety and *Cg*1 on the symmetry related neighbouring molecule with symmetry code: (ii) *x*, -*y*, *z* - 1/2. Figure 2 shows the packing within the unit cell for the title compound.

### Experimental

The compound was synthesized following a literature method (Pal *et al.*, 1992). Under a high flow of nitrogen, 6 ml of 20% NaOH solution was added to an aqueous solution of 2-picolyl chloridehydrochloride [(3.937 g (24 mmol) in 0.5 ml ultra pure water] to form a pink emulsion solution. 2-Amino-2-methyl propane (12 mmol) was added and the mixture stirred at 60°C. 40 ml of 20% NaOH solution was then added over a period of 1 h and the mixture left to stir for a further 12 h. The crude product was extracted with CHCl_3_ washed with ultra pure water and dried over Na_2_SO_4_. Excess solvent was removed under reduced pressure and the oil residue purified on a short chromatographic column packed with 0.5 g charcoal and 5 g of neutral alumina using CHCl_3_ as an eluent to afford a light yellow solution. Colourless single crystals suitable for X-ray diffraction were obtained from slow evaporation of the solvent from its solution made from a 5% chloroform in ethanol solution (yield: 1.73 g, 60%).

Spectroscopic data: ^1^H NMR (400 MHz, CDCl_3_) δ / p.p.m.: 8.40 (d, 2H), 7.60 (t, 2H), 7.45 (d, 2H), 7.05 (t, 2H), 3.95 (s, 4H), 1.18 (s, 9H). ^13^C NMR (125 MHz, CDCl_3_) δ / p.p.m.: 27.0, 57.0, 122.0, 123.0,136.0, 148.0, 160. Anal. Calc. for C_16_H_21_N_3_: C 75.26, H 8.29, N 16.46; Found: C 74.89, H 7.97, N 17.37.

### Refinement

All Hydrogen atoms were positioned in geometrically idealized positions and constrained to ride on their parent atoms with C—H distances in the range 0.93–0.97 Å, and with *U*_iso_(H) = 1.2 or 1.5*U*_eq_(C). In the absence of significant anomalous scattering effects, Friedel pairs have been merged.

### Crystal data

**Table:**

C_16_H_21_N_3_	*F*(000) = 552
*M**_r_* = 255.36	*D*_x_ = 1.133 Mg m^−^^3^
Monoclinic, *C**c*	Mo *K*α radiation, λ = 0.71073 Å
Hall symbol: C -2yc	Cell parameters from 4672 reflections
*a* = 6.1808 (3) Å	θ = 3.9–32.0°
*b* = 17.9502 (8) Å	µ = 0.07 mm^−^^1^
*c* = 13.7079 (6) Å	*T* = 293 K
β = 100.239 (4)°	Plate, colourless
*V* = 1496.62 (12) Å^3^	0.50 × 0.50 × 0.30 mm
*Z* = 4	

### Data collection

**Table:**

Oxford Diffraction Xcalibur2 CCD diffractometer	2392 independent reflections
Radiation source: Enhance (Mo)X-Ray Source	2024 reflections with *I* > 2σ(*I*)
graphite	*R*_int_ = 0.012
Detector resolution: 8.4190 pixels mm^-1^	θ_max_ = 31.9°, θ_min_ = 4.1°
ω–2θ scans	*h* = −8→7
Absorption correction: multi-scan (*CrysAlis RED*; Oxford Diffraction, 2008)	*k* = −26→26
*T*_min_ = 0.967, *T*_max_ = 0.980	*l* = −18→20
7475 measured reflections	

### Refinement

**Table:**

Refinement on *F*^2^	Primary atom site location: structure-invariant direct methods
Least-squares matrix: full	Secondary atom site location: difference Fourier map
*R*[*F*^2^ > 2σ(*F*^2^)] = 0.043	Hydrogen site location: inferred from neighbouring sites
*wR*(*F*^2^) = 0.119	H-atom parameters constrained
*S* = 1.02	*w* = 1/[σ^2^(*F*_o_^2^) + (0.0873*P*)^2^ + 0.017*P*] where *P* = (*F*_o_^2^ + 2*F*_c_^2^)/3
2392 reflections	(Δ/σ)_max_ = 0.001
175 parameters	Δρ_max_ = 0.21 e Å^−^^3^
2 restraints	Δρ_min_ = −0.21 e Å^−^^3^
0 constraints	

### Special details

**Table:**

Geometry. All e.s.d.'s (except the e.s.d. in the dihedral angle between two l.s. planes) are estimated using the full covariance matrix. The cell e.s.d.'s are taken into account individually in the estimation of e.s.d.'s in distances, angles and torsion angles; correlations between e.s.d.'s in cell parameters are only used when they are defined by crystal symmetry. An approximate (isotropic) treatment of cell e.s.d.'s is used for estimating e.s.d.'s involving l.s. planes.
Refinement. Refinement of *F*^2^ against ALL reflections. The weighted *R*-factor *wR* and goodness of fit *S* are based on *F*^2^, conventional *R*-factors *R* are based on *F*, with *F* set to zero for negative *F*^2^. The threshold expression of *F*^2^ > σ(*F*^2^) is used only for calculating *R*-factors(gt) *etc*. and is not relevant to the choice of reflections for refinement. *R*-factors based on *F*^2^ are statistically about twice as large as those based on *F*, and *R*- factors based on ALL data will be even larger.

### Fractional atomic coordinates and isotropic or equivalent isotropic displacement parameters (Å^2^)

**Table:**

	*x*	*y*	*z*	*U*_iso_*/*U*_eq_	
C14	0.2251 (5)	0.16330 (11)	0.59751 (18)	0.0645 (6)	
H20	0.1452	0.1875	0.6425	0.097*	
H21	0.2314	0.1955	0.5421	0.097*	
H19	0.3716	0.1525	0.6311	0.097*	
C15	0.2342 (5)	0.05704 (14)	0.48509 (17)	0.0679 (6)	
H18	0.3767	0.0409	0.5180	0.102*	
H16	0.2508	0.0937	0.4359	0.102*	
H17	0.1537	0.0152	0.4537	0.102*	
C16	−0.1269 (5)	0.10773 (16)	0.5103 (2)	0.0739 (7)	
H15	−0.1940	0.0630	0.4806	0.111*	
H14	−0.1260	0.1449	0.4600	0.111*	
H13	−0.2090	0.1258	0.5586	0.111*	
N3	0.1632 (2)	0.14031 (8)	0.88126 (11)	0.0448 (3)	
C9	0.2329 (2)	0.09809 (8)	0.81277 (11)	0.0365 (3)	
N1	0.1221 (2)	0.03781 (7)	0.64559 (9)	0.0382 (3)	
C3	0.1172 (3)	−0.09657 (8)	0.68278 (12)	0.0384 (3)	
C10	0.4530 (3)	0.08012 (10)	0.81845 (13)	0.0434 (3)	
H8	0.4982	0.0513	0.7694	0.052*	
C1	0.1094 (3)	0.09104 (10)	0.56073 (13)	0.0455 (4)	
C2	0.0134 (3)	−0.03384 (10)	0.61861 (13)	0.0452 (4)	
H11	0.0200	−0.0449	0.5499	0.054*	
H12	−0.1403	−0.0300	0.6245	0.054*	
N2	−0.0196 (2)	−0.14516 (8)	0.71406 (13)	0.0476 (3)	
C8	0.0527 (3)	0.06774 (11)	0.73393 (12)	0.0442 (4)	
H9	−0.0232	0.0287	0.7633	0.053*	
H10	−0.0527	0.1073	0.7137	0.053*	
C12	0.5344 (3)	0.14791 (10)	0.96934 (14)	0.0529 (4)	
H6	0.6329	0.1652	1.0240	0.063*	
C11	0.6047 (3)	0.10564 (11)	0.89812 (15)	0.0501 (4)	
H7	0.7530	0.0941	0.9031	0.060*	
C4	0.3437 (3)	−0.10609 (10)	0.70322 (14)	0.0466 (4)	
H4	0.4352	−0.0713	0.6809	0.056*	
C13	0.3140 (4)	0.16421 (10)	0.95790 (14)	0.0519 (4)	
H5	0.2664	0.1935	1.0060	0.062*	
C5	0.4322 (3)	−0.16716 (12)	0.75666 (16)	0.0553 (5)	
H3	0.5836	−0.1743	0.7708	0.066*	
C6	0.2920 (4)	−0.21757 (11)	0.78883 (17)	0.0567 (5)	
H2	0.3460	−0.2594	0.8252	0.068*	
C7	0.0701 (3)	−0.20409 (10)	0.76555 (17)	0.0554 (5)	
H1	−0.0242	−0.2383	0.7871	0.066*	

### Atomic displacement parameters (Å^2^)

**Table:**

	*U*^11^	*U*^22^	*U*^33^	*U*^12^	*U*^13^	*U*^23^
C14	0.0927 (17)	0.0403 (9)	0.0584 (12)	−0.0074 (10)	0.0077 (11)	0.0060 (8)
C15	0.0990 (18)	0.0620 (12)	0.0486 (11)	−0.0019 (12)	0.0296 (11)	0.0014 (9)
C16	0.0720 (14)	0.0773 (16)	0.0638 (13)	0.0155 (12)	−0.0114 (11)	0.0176 (11)
N3	0.0476 (8)	0.0460 (7)	0.0408 (7)	0.0030 (6)	0.0075 (6)	−0.0021 (5)
C9	0.0370 (7)	0.0395 (7)	0.0324 (6)	−0.0014 (5)	0.0047 (5)	0.0033 (5)
N1	0.0426 (7)	0.0397 (6)	0.0307 (6)	−0.0034 (5)	0.0021 (5)	0.0008 (4)
C3	0.0404 (8)	0.0389 (7)	0.0347 (7)	−0.0029 (6)	0.0033 (5)	−0.0005 (5)
C10	0.0369 (7)	0.0529 (9)	0.0396 (7)	0.0002 (6)	0.0044 (6)	−0.0019 (6)
C1	0.0559 (10)	0.0416 (8)	0.0371 (7)	0.0006 (6)	0.0034 (7)	0.0054 (6)
C2	0.0446 (8)	0.0451 (8)	0.0409 (8)	−0.0067 (6)	−0.0060 (6)	0.0039 (6)
N2	0.0412 (7)	0.0437 (7)	0.0568 (9)	−0.0058 (6)	0.0056 (6)	0.0045 (6)
C8	0.0342 (7)	0.0603 (10)	0.0369 (7)	−0.0018 (6)	0.0031 (6)	−0.0030 (6)
C12	0.0612 (11)	0.0493 (9)	0.0427 (9)	−0.0136 (8)	−0.0054 (8)	0.0022 (7)
C11	0.0403 (8)	0.0573 (10)	0.0493 (9)	−0.0036 (7)	−0.0013 (7)	0.0072 (7)
C4	0.0371 (8)	0.0526 (9)	0.0514 (9)	−0.0026 (6)	0.0114 (7)	0.0003 (7)
C13	0.0678 (11)	0.0456 (8)	0.0413 (9)	−0.0018 (8)	0.0071 (8)	−0.0066 (7)
C5	0.0433 (9)	0.0576 (10)	0.0637 (12)	0.0114 (8)	0.0061 (8)	−0.0027 (9)
C6	0.0618 (11)	0.0419 (8)	0.0640 (11)	0.0110 (7)	0.0043 (9)	0.0033 (8)
C7	0.0539 (10)	0.0426 (9)	0.0702 (12)	−0.0057 (7)	0.0127 (9)	0.0088 (8)

### Geometric parameters (Å, °)

**Table:**

C14—C1	1.523 (3)	C3—C2	1.501 (2)
C14—H20	0.9600	C10—C11	1.385 (2)
C14—H21	0.9600	C10—H8	0.9300
C14—H19	0.9600	C2—H11	0.9700
C15—C1	1.525 (3)	C2—H12	0.9700
C15—H18	0.9600	N2—C7	1.336 (2)
C15—H16	0.9600	C8—H9	0.9700
C15—H17	0.9600	C8—H10	0.9700
C16—C1	1.531 (3)	C12—C11	1.367 (3)
C16—H15	0.9600	C12—C13	1.375 (3)
C16—H14	0.9600	C12—H6	0.9300
C16—H13	0.9600	C11—H7	0.9300
N3—C9	1.336 (2)	C4—C5	1.377 (3)
N3—C13	1.345 (2)	C4—H4	0.9300
C9—C10	1.387 (2)	C13—H5	0.9300
C9—C8	1.509 (2)	C5—C6	1.378 (3)
N1—C8	1.457 (2)	C5—H3	0.9300
N1—C2	1.468 (2)	C6—C7	1.374 (3)
N1—C1	1.497 (2)	C6—H2	0.9300
C3—N2	1.338 (2)	C7—H1	0.9300
C3—C4	1.388 (2)		
			
C1—C14—H20	109.5	C15—C1—C16	109.18 (19)
C1—C14—H21	109.5	N1—C2—C3	112.38 (12)
H20—C14—H21	109.5	N1—C2—H11	109.1
C1—C14—H19	109.5	C3—C2—H11	109.1
H20—C14—H19	109.5	N1—C2—H12	109.1
H21—C14—H19	109.5	C3—C2—H12	109.1
C1—C15—H18	109.5	H11—C2—H12	107.9
C1—C15—H16	109.5	C7—N2—C3	117.31 (16)
H18—C15—H16	109.5	N1—C8—C9	116.05 (14)
C1—C15—H17	109.5	N1—C8—H9	108.3
H18—C15—H17	109.5	C9—C8—H9	108.3
H16—C15—H17	109.5	N1—C8—H10	108.3
C1—C16—H15	109.5	C9—C8—H10	108.3
C1—C16—H14	109.5	H9—C8—H10	107.4
H15—C16—H14	109.5	C11—C12—C13	118.10 (17)
C1—C16—H13	109.5	C11—C12—H6	121.0
H15—C16—H13	109.5	C13—C12—H6	121.0
H14—C16—H13	109.5	C12—C11—C10	119.36 (17)
C9—N3—C13	117.70 (16)	C12—C11—H7	120.3
N3—C9—C10	121.88 (15)	C10—C11—H7	120.3
N3—C9—C8	114.75 (14)	C5—C4—C3	119.78 (16)
C10—C9—C8	123.24 (14)	C5—C4—H4	120.1
C8—N1—C2	110.06 (14)	C3—C4—H4	120.1
C8—N1—C1	115.35 (14)	N3—C13—C12	123.79 (17)
C2—N1—C1	113.90 (12)	N3—C13—H5	118.1
N2—C3—C4	121.83 (15)	C12—C13—H5	118.1
N2—C3—C2	116.63 (15)	C4—C5—C6	118.68 (17)
C4—C3—C2	121.42 (15)	C4—C5—H3	120.7
C11—C10—C9	119.16 (17)	C6—C5—H3	120.7
C11—C10—H8	120.4	C7—C6—C5	117.95 (18)
C9—C10—H8	120.4	C7—C6—H2	121.0
N1—C1—C14	109.25 (14)	C5—C6—H2	121.0
N1—C1—C15	108.06 (15)	N2—C7—C6	124.45 (18)
C14—C1—C15	107.53 (19)	N2—C7—H1	117.8
N1—C1—C16	112.96 (17)	C6—C7—H1	117.8
C14—C1—C16	109.70 (18)		
			
C13—N3—C9—C10	−1.1 (2)	C2—C3—N2—C7	175.54 (17)
C13—N3—C9—C8	174.86 (16)	C2—N1—C8—C9	134.62 (15)
N3—C9—C10—C11	1.0 (2)	C1—N1—C8—C9	−94.82 (18)
C8—C9—C10—C11	−174.62 (17)	N3—C9—C8—N1	165.09 (14)
C8—N1—C1—C14	50.8 (2)	C10—C9—C8—N1	−19.0 (2)
C2—N1—C1—C14	179.45 (17)	C13—C12—C11—C10	−0.8 (3)
C8—N1—C1—C15	167.47 (17)	C9—C10—C11—C12	0.0 (3)
C2—N1—C1—C15	−63.8 (2)	N2—C3—C4—C5	0.3 (3)
C8—N1—C1—C16	−71.6 (2)	C2—C3—C4—C5	−175.51 (17)
C2—N1—C1—C16	57.1 (2)	C9—N3—C13—C12	0.3 (3)
C8—N1—C2—C3	−77.86 (18)	C11—C12—C13—N3	0.7 (3)
C1—N1—C2—C3	150.82 (15)	C3—C4—C5—C6	−0.1 (3)
N2—C3—C2—N1	137.29 (16)	C4—C5—C6—C7	0.1 (3)
C4—C3—C2—N1	−46.7 (2)	C3—N2—C7—C6	0.5 (3)
C4—C3—N2—C7	−0.5 (3)	C5—C6—C7—N2	−0.3 (3)

### Hydrogen-bond geometry (Å, °)

**Table:**

*D*—H···*A*	*D*—H	H···*A*	*D*···*A*	*D*—H···*A*
C7—H1···Cg2^i^	0.93	2.97	3.819 (2)	153
C15—H16···Cg1^ii^	0.96	2.94	3.836 (2)	156

Symmetry codes: (i) *x*−1/2, *y*−1/2, *z*; (ii) *x*, −*y*, *z*−1/2.
